# Is PTEN loss associated with clinical outcome measures in human prostate cancer?

**DOI:** 10.1038/sj.bjc.6604680

**Published:** 2008-10-14

**Authors:** P McCall, C J Witton, S Grimsley, K V Nielsen, J Edwards

**Affiliations:** 1 Section of Surgery, Division of Cancer Sciences and Molecular Pathology, University of Glasgow, Glasgow Royal Infirmary, Glasgow G31 3ER, UK; 2 Department of Molecular Pathology, Dako A/S, Glostrup, Denmark

**Keywords:** PTEN, Akt, prostate, hormone refractory

## Abstract

Inactivating PTEN mutations are commonly found in prostate cancer, resulting in an increased activation of Akt. In this study, we investigate the role of PTEN deletion and protein expression in the development of hormone-refractory prostate cancer using matched hormone-sensitive and hormone-refractory tumours. Fluorescent *in situ* hybridisation and immunohistochemistry was carried out to investigate *PTEN* gene deletion and PTEN protein expression in the transition from hormone-sensitive to hormone-refractory prostate cancer utilising 68 matched hormone sensitive and hormone-refractory tumour pairs (one before and one after hormone relapse). Heterogeneous *PTEN* gene deletion was observed in 23% of hormone sensitive tumours. This increased significantly to 52% in hormone-refractory tumours (*P*=0.044). PTEN protein expression was observed in the membrane, cytoplasm and the nucleus. In hormone sensitive tumours, low levels of cytoplasmic PTEN was independently associated with shorter time to relapse compared to high levels of PTEN (*P*=0.028, hazard ratio 0.51 (95%CI 0.27–0.93). Loss of PTEN expression in the nucleus of hormone sensitive tumours was independently associated with disease-specific survival (*P*=0.031, hazard ratio 0.52, 95%CI 0.29–0.95). The results from this study demonstrate a role for both cytoplasmic and nuclear PTEN in progression of prostate cancer to the hormone-refractory state.

Every year in the Unite Kingdom, almost 32 000 cases of prostate cancer are diagnosed and approximately 10 000 die of the disease (Cancer Research UK web site, 2004). The underlying molecular mechanisms of prostate cancer initiation and progression are largely unknown because of extensive tumour heterogeneity. Patients diagnosed with locally advanced or metastatic prostate cancer may be treated by androgen ablation therapy, resulting in a reduction of androgens in the circulation and inhibition of tumour growth. This treatment is initially successful, but patients tend to relapse within 18–24 months with disease progression refractory to therapy (Arnold and Isaacs, 2002). Hormone-refractory prostate cancer has a poor prognosis, with median survival period reported to be 24 months. Loss of androgen dependence is often correlated with the overexpression of antiapoptotic and cell survival signalling pathways (Johnson and Hamdy, 1998; Karan *et al*, 2003). Components of these pathways are mutated or abnormally expressed in many tumour types and mutations of upstream receptors, such as EGFR, transmit their signals through these cascades. The loss of tumour suppressor function is also a common event in the progression to hormone-refractory prostate cancer and is associated with the gain of oncogenic signalling (Isaacs and Kainu, 2001). P53, retinablastoma (Rb), NKX3.1 and phosphatase and tensin homologue deleted on chromosome 10 (PTEN) have all been well documented to have a loss of function during the progression to hormone-refractory prostate cancer (Bookstein *et al*, 1990; Phillips *et al*, 1994; He *et al*, 1997; Davies *et al*, 1999; McMenamin *et al*, 1999).

Phosphatase and tensin homologue deleted on chromosome 10 functions as a lipid phosphatase that dephosporylates phosphatidylinositol (3, 4 and 5) triphosphate (PIP3), a second messenger of PI3K at the plasma membrane. PIP3 recruits phosphatidylinositol-dependent kinase-1 (PDK1) and Akt to the membrane. Here, PDK1 phosphorylates Akt, which leads to its activation. Akt mediates signals downstream that promote cell survival and proliferation. Phosphatase and tensin homologue deleted on chromosome 10 mutants that retain protein tyrosine phosphatase activity, but lose the ability to dephosphorylate PIP3, are found in many tumours, indicating that the lipid phosphatase activity is needed for tumour suppression (Myers *et al*, 1998). To function in this manner, PTEN must be located in the cytoplasm; however, it has recently emerged that there is also a role for PTEN in the nucleus (Eng, 2003; Chung and Eng, 2005; Lian and Di Cristofano, 2005).

Prostate cancer cell lines that have been cultured from metastatic sites, such as the lymph nodes (LNCaP) or brain metastasis (PC3) have highly active PI3K/Akt signalling (Davies *et al*, 1999; Murillo *et al*, 2001). *PTEN* mutation has been associated with 5–27% of localised and 30–60% of metastatic prostate tumours (Feilotter *et al*, 1998; Suzuki *et al*, 1998; Wang *et al*, 1998). In addition, loss of PTEN expression is associated with disease progression and increased risk of recurrence (Gray *et al*, 1998; McMenamin *et al*, 1999; Burton *et al*, 2000; Reiss *et al*, 2000; Fenci and Woenckhaus, 2002), although substantial heterogeneity has been observed between different metastatic sites within the same patients (Suzuki *et al*, 1998). Here, we examined both the deletion of the *PTEN* gene and expression of PTEN protein at individual cellular locations in a cohort of matched hormone-sensitive and hormone-refractory prostate tumours, with the aim of clarifying the prognostic significance of PTEN loss in prostate cancer. Using the same cohort, we have previously demonstrated that the upregulation of Akt activation is significantly associated with decreased survival, and therefore PTEN loss is one possible route by which this may occur (McCall *et al*, 2008).

## Materials and methods

### Patients

Sixty-eight patients with matched hormone-sensitive and hormone-refractory tumour pairs were retrospectively selected for the analysis. All tumours had patient identification removed, and the clinical information database was anonymised. Ethical approval was obtained from the Multicentre Research Ethics Committee for Scotland (MREC/01/0/36) and fron the Local Research and Ethical Committees. Patients were only selected for analysis if they initially responded to hormone treatment (in the form of subcapsular bilateral orchidectomy or maximum androgen blockade), but subsequently relapsed (two consecutive rises in PSA greater than 10%) and had a pre- and post-hormone relapse tissue sample available for analysis. Hormone-refractory prostate cancer specimens were obtained by TURP, when patients required further surgical procedures to treat clinical symptoms such as bladder outflow obstruction. All these samples were obtained with 8 weeks of biochemical relapse being diagnosed (two consecutive rises in PSA greater than 10%). Phosphorylated Akt expression is already available for this patient cohort (McCall *et al*, 2008).

### Fluorescent *in situ* hybridisation

**Figure 1:**
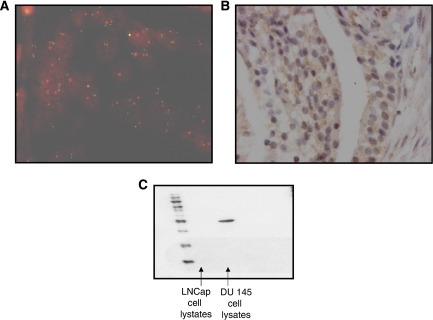
(**A**) shows an example of fluorescent *in situ* hybridisation for chromosome 10 (red signal) and *PTEN* (green signal). (**B**) Shows an example of immunohistochemistry for PTEN protein expression; in hormone-sensitive prostate cancer, both cytoplasmic expression and nuclear expression are present. (**C**) Western blot analysis for PTEN protein to confirm antibody specificity. LNCaP cells do not express PTEN protein, whereas DU145 cells do express PTEN protein. Lane 1, hormone-sensitive LNCaP cell lysates; lane 2, hormone-refractory LNCaP cell lysates; lane 3, DU145 cell lysates.

Fluorescent *in situ* hybridisation (FISH) was performed on 5 *μ*m, archival formalin fixed, paraffin-embedded prostate tumour tissue arrays. Slides were incubated for 1 h at 56°C, dewaxed and rehydrated through graded alcohols. Tissue was then pretreated using histology FISH accessory kit (Dako A/S, Glostrup, Denmark). In brief, slides were rinsed twice in wash buffer and then incubated for 10 min in pretreatment buffer at 95°C, followed by a 3-min incubation at room temperature in wash buffer. Slides were then incubated in pepsin for 26 min at 25°C, followed by a 3-min incubation at room temperature in wash buffer. Dako A/S provided *PTEN* probe; 10 *μ*l of probe was applied to each slide and incubated at 82°C for 22 min followed by an overnight incubation at 45°C. Slides were then washed in stringent wash buffer for 10 min at 65°C, followed by two 3-min washes in wash buffer. Slides were dehydrated through graded alcohols, mounted in DAPI Vectashield (Vector Laboratories, CA, USA) and viewed using a Leica DMLB microscope at × 400 magnification (Figure 1A). Fluorescent *in situ* hybridisation was scored by two independent observers; the number of signals for each chromosome (green) and gene (red) were counted in 20 non-overlapping nuclei. The gene-to-chromosome ratio was then calculated. *PTEN* loss was classified as a gene-to-chromosome ratio of ⩽0.8 (Watters *et al*, 1998).

### Immunohistochemistry

Tumour expression of PTEN was determined in archival formalin fixed, paraffin-embedded prostate tissue sections (5 *μ*m) by IHC. Sections were dewaxed in xylene and rehydrated through graded alcohols before blocking endogenous peroxidase in 3% hydrogen peroxide. Antigen retrieval was performed by heating tissue sections under pressure in citrate buffer (pH 6) for 5 min. Sections were blocked using 1.5% horse serum; PTEN antibody (Cell Signalling Technology) was used at 1 *μ*g ml^−1^, and incubated overnight at 4°C. Staining was developed using Envision plus kit (Dako A/S). Nuclei were counterstained with haematoxylin before mounting. An example of the staining is shown in Figure 1 (Figure 1B). A positive and negative control slide was included in each IHC run; negative controls were incubated with an isotype matched control antibody at a concentration of 1 mg ml^−1^. Cell pellets known to express or not express PTEN were also included in each run. Antibody specificity was confirmed by western blot analysis (Figure 1C).

Staining was scored blind by two independent observers using a weighted histoscore method (Kirkegaard *et al*, 2006) also known as the H-score system (McCarty *et al*, 1986). Histoscores were calculated from the sum of (1 × the percentage of cells staining weakly positive) + (2 × the percentage of cells staining moderately positive) + (3 × the percentage of cells staining strongly positive) with a maximum histoscore of 300. The interclass correlation coefficient (ICCC) between each observer was confirmed to measure consistency. The ICCC value was >0.7, which is classed as excellent as; an ICCC of 1 indicates identical score (Kirkegaard *et al*, 2006). The mean of the two observer's histoscores was used for the analysis. Changes in protein-expression staining between hormone-sensitive and hormone-refractory cases were defined as an increase or decrease with the 95% confidence interval for the difference in the interobserver variation (ie, the mean difference between the histoscore that each observer assigns for protein expression ±2 s.d.; Edwards *et al*, 2003).

All statistical analyses were performed using the SPSS version 9.0 for Windows. Protein expression data were not normally distributed and is given as median and interquartile ranges. Wilcoxon signed-rank tests were used to compare protein expression between hormone-sensitive and hormone-refractory prostate cancer tumours. Survival analysis was conducted using the Kaplan–Meier method, and curves were compared with the log-rank test. Multivariate survival analysis and hazard ratios (HRs) were calculated using Cox regression analysis. A value of *P*<0.05 was considered statistically significant.

## Results

Patients in this cohort were diagnosed with locally advanced (50) or metastatic prostate cancer (18), and subsequently received surgery and androgen deprivation therapy (26 subcapsular bilateral orchidectomy, 44 GnRH analogue and two had both). In all, 45 of the 68 patients also received an antiandrogen therapy and this included all those who received GnRH analogues. At initial diagnosis, the median age was 70 (66–74) years and 26% of patients had metastatic disease. The median time to biochemical relapse was 2.32 (1.48–4.00) years and the percentage of patients with metastatic disease had increased to 57%. Sixty-one patients (89.7%) died during follow-up, and median survival for these patients was 4.34 (2.94–6.63) years. Seven patients were alive at the last follow-up; the median time of follow-up for all 68 patients was 4.34 (2.86–6.74) years.

### Factors associated with time to biochemical relapse, time to death from biochemical relapse and disease-specific survival

When serum PSA level, age, metastasis and Gleason grade at diagnosis were analysed for this patient, cohort using the Kaplan–Meier log-rank method, PSA at diagnosis (*P*=0.036) and Gleason score at diagnosis (*P*=0.010) were associated with shorter time to biochemical relapse.

Death at the time of biochemical relapse was associated with PSA level at relapse (*P*=0.016). Overall survival was associated with the presence of metastases at relapse (*P*=0.0019) and Gleason score at diagnosis (*P*=0.049).

### Fluorescent *in situ* hybridisation

#### PTEN deletions

Of the 68 prostate carcinomas investigated, *PTEN*/chromosome 10 ratio was successfully evaluated in 57 (84%) cases. The remaining cases were excluded from the study because of insufficient tumour material in the cores. The mean *PTEN*/chromosome 10 ratio for the hormone-sensitive and hormone-refractory tumours was 0.98 (range: 0.71–1.11) and 0.92 (range: 0.39–2.16), respectively. Gene deletion as identified by FISH was observed in 23% of hormone-sensitive tumours; this increased significantly to 52% in hormone-refractory tumours (*P*=0.044). Loss of one copy of *PTEN* was commonly observed, and this was heterogeneous in nature, being frequently observed in only one area of tumour. Loss of *PTEN* was correlated with prostate cancer progression; however, no correlation was observed between the loss of *PTEN* and Gleason score at diagnosis, loss of *PTEN* and presence of metastasis at diagnosis or the loss of *PTEN* and PSA at diagnosis. When loss of *PTEN* was correlated with survival, a trend between the loss of *PTEN* and poorer disease-specific survival was noted (*P*=0.086); this was not independently significant by Cox regression analysis.

#### Gene number and chromosomal aneusomy

In the informative cases, the mean *PTEN* gene copy number per counted cancer cell for the hormone-sensitive and hormone-refractory cases was 1.90 (range: 1.4–2.5) and 1.71 (range: 0.80–3.45) respectively. When assessing the frequency of chromosome aneusomy, the mean chromosomal copy number of chromosome 10 for the hormone-sensitive and hormone-refractory tumours was 1.93 (range: 1.75–2.25) and 1.87 (range: 1.60–2.05), respectively. Normal range for chromosomal copy number is 1.35–2.01, in this cohort, none of the hormone-sensitive or hormone-refractory tumours appeared to have lost chromosome 10 as 0% had chromosome 10 copy number per counted cancer cell of less than 1.35, which is the lower limit of the normal range. In contrast, 12% of the hormone-sensitive tumours and 21% of the hormone-refractory tumours had chromosome 10 copy numbers per counted cancer cell higher than that of the normal range. It was noted that this was a different subgroup of patients exhibiting the loss of *PTEN* gene.

### Immunohistochemistry

Membrane, cytoplasmic and nuclear PTEN expression was observed in prostate tissue. In the hormone-sensitive tumours, membrane and nuclear expression were less frequently observed than cytoplasmic expression; 41 and 46% of patients, respectively, had membrane and nuclear expression compared with 95% expressing PTEN in the cytoplasm. The interquartile range of expression for each location is shown in Table 1. This rate of expression did not significantly change in the hormone-refractory tissue. When median protein expression levels in the hormone-sensitive and hormone-refractory tissue were compared, no statistically significant change was observed at any cellular location. Loss of PTEN expression was heterogeneous.

### Hormone-sensitive tumours

**Figure 2:**
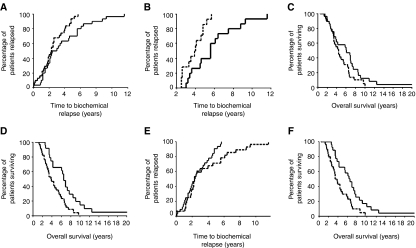
(**A**) Shows a Kaplan–Meier plot for high (above the median, solid line) and low (below the median, dotted line) PTEN cytoplasmic expression and time to biochemical relapse (*P*=0.027). (**B**) Shows a Kaplan–Meier plot for high (above the median, solid line) and low (below the median, dotted line) PTEN cytoplasmic expression for patients that took longer than 30 months to relapse (*P*=0.0035). (**C**) shows a Kaplan–Meier plot for high (above the median, solid line) and low (below the median, dotted line) PTEN cytoplasmic expression and disease-specific survival (labelled overall survival; *P*=0.072). (**D**) Shows a Kaplan–Meier plot for patients with tumours that have membrane PTEN expression (solid line) compared to patients whose tumours do not have nuclear PTEN expression (dotted line) and disease-specific survival (labelled overall survival; *P*=0.002). (**E**) Shows a Kaplan–Meier plot for patients with tumours that have nuclear PTEN expression (dotted line) compared to patients whose tumours do not have nuclear PTEN expression (solid line) and time to biochemical relapse (*P*=0.078). (**F**) Shows a Kaplan–Meier plot for patients with tumours that have nuclear PTEN expression (solid line) compared to patients whose tumours do not have nuclear PTEN expression (dotted line) and disease-specific survival (labelled overall survival; *P*=0.003).

To determine whether protein expression was linked to time to biochemical relapse, Kaplan–Meier graphs were plotted for the hormone-sensitive tumours expressing low levels of protein (<median histoscore) *vs* high levels of protein (>median histoscore), and compared using the log-rank test. The patients whose tumours expressed low levels of PTEN in the cytoplasm were shown to have relapsed significantly earlier than those patients whose tumours expressed high levels of PTEN in the cytoplasm (Figure 2A, *P*=0.027). Cox regression analysis indicates that cytoplasmic PTEN expression is independent of known clinical prognostic factors (*P*=0.028, HR 0.51 (95% CI 0.27–0.93). It was noted, however, that the Kaplan–Meier curves did not separate until approximately 2.5 years after diagnosis. Therefore, PTEN loss appeared to be influencing relapse in those patients who took more than 30 months to relapse. If patients who relapsed within 30 months were excluded from the analysis, the median time to relapse for those with low PTEN expression was 3.9 (IQR 2.98–4.92) years compared to 5.6 (4.36–6.84) years for those with high PTEN expression (*P*=0.0035; Figure 2B). In addition, those patients with high levels of cytoplasmic PTEN expression in their hormone-sensitive tumours were observed to have longer median overall survival (6.1 years (IQR 2.8–9.4)) compared to those with low PTEN expression (4.4 years (IQR 3.3–5.4)), although this did not reach significance (*P*=0.072; Figure 2C). Again the curves first separate approximately 30 months after diagnosis.

Expression of phosphorylated Akt at serine 473 (activated Akt) for this cohort of patients had already been established in a previous study (McCall *et al*, 2008). Levels of phosphorylated Akt expression in tumours that expressed low levels of PTEN was higher compared to tumours that expressed high levels of PTEN (*P*=0.047).

The median for both PTEN membrane and nuclear expression in the hormone-sensitive tumours was 0; therefore patients were divided into those patients whose tumours did not express PTEN at these locations and those that did. Patients with PTEN membrane expression in their hormone-sensitive tumour had significantly longer overall survival than those patients without (Figure 2D, *P*=0.002). The median time to relapse for patients whose tumours did not express PTEN in the membrane was 3.8 years (IQR 2.7–4.85) compared to 6.5 (IQR 5.8–7.2) years for patients with membrane expression.

Patients with no nuclear PTEN expression relapsed earlier than those with nuclear PTEN expression, although this did not reach significance (Figure 2E, *P*=0.078). PTEN nuclear expression was associated with overall survival, those patients whose tumours had no nuclear PTEN expression had a significantly shorter overall survival compared to those patients with PTEN nuclear expression (Figure 2F, *P*=0.003). Median overall survival was 3.4 years (IQR 2.6–4.2) compared to 6.5 years (IQR 5.1–7.8) that confers a survival advantage of 3 years for those patients whose tumours express nuclear PTEN. Nuclear PTEN expression was demonstrated to be an independent prognostic marker by Cox regression analysis, when compared with known clinical prognostic parameters (*P*=0.031, HR 0.52 (95% CI 0.29–0.95).

As observed with membrane expression, phosphorylated Akt expression was lower in the nucleus of tumours with high levels of nuclear PTEN than those with low levels, but this did not reach significance (*P*=0.132). In contrast, PTEN membrane expression correlated strongly with nuclear PTEN expression (*P*<0.001, *R*_S_ 0.66).

### Hormone-refractory tumours

Phosphatase and tensin homologue deleted on chromosome 10 expression levels in the hormone-refractory tumours were not associated with time to death from relapse, disease-specific death from relapse or with the presence of metastasis.

## Discussion

The *PTEN* tumour suppressor has emerged as a critical regulator of cellular processes, which is frequently mutated or deleted in a number of human cancers, including prostate cancer. Fluorescent *in situ* hybridisation and IHC have demonstrated that a significant proportion of patients have heterogeneous *PTEN* deletion and loss of PTEN protein expression, which is, associated with clinical outcome measures.

The frequency and mode of PTEN inactivation reported at various stages of clinical prostate cancer are variable (Verhagen *et al*, 2006). In this study, we investigated the level of *PTEN* loss by FISH in matched hormone-sensitive and hormone-refractory tumours. Loss was noted in 23% hormone-sensitive tumours compared with 52% hormone-refractory tumours; these rates are similar to those previously reported by FISH analysis (Verhagen *et al*, 2006). Fluorescent *in situ* hybridisation depending on the region that the probe binds to does not always detect small deletions, and in the case of our study, the probe covers the whole of the *PTEN* gene. Therefore, in this study, loss of the whole gene is being measured. It was observed that very few tumours had homogeneous *PTEN* deletion and complete loss of PTEN expression (2%), but almost all have heterogeneous loss of expression; this is consistent with previous reports (Verhagen *et al*, 2006). In this study, PTEN loss does not correlate with *PTEN* gene deletion, although all tumours with *PTEN* deletion have low PTEN expression. An explanation for low PTEN expression in tumours that appear not to have *PTEN* deletion is the hypermethylation of the *PTEN* promoter region. Evidence for promoter hypermethylation has been reported in prostate cancer xenografts (Whang *et al*, 1998). Although the mechanism of PTEN inactivation is currently controversial and possibly because of different mechanisms in different tumours, it is widely accepted that *PTEN* loss is one of the most common events associated with prostate cancer (Majumder and Sellers, 2005). Consistent with these findings, this study observed a higher rate of *PTEN* deletion by FISH in hormone-refractory compared to hormone sensitive tumours, suggesting that *PTEN* loss is associated with tumour progression. Although this was not significantly associated with clinical outcome measures, a trend was observed demonstrating that those patients with *PTEN* loss had shorter overall survival. If the FISH studies were expanded to a larger data set, these results might have reached significance.

As predicted, loss or low cytoplasmic PTEN expression was independently associated with time to relapse and linked with increased Akt activation. Surprisingly, however, this was observed to be a late event with curves separating 30 months following diagnosis, suggesting that other factors, such as PI3K expression, may also contribute to Akt activation and disease progression. Cytoplasmic PTEN expression was only weakly associated with overall survival, and this did not reach significance in this study.

However, in addition to cytoplasmic PTEN expression, nuclear PTEN expression was also observed. Unlike cytoplasmic PTEN expression, loss of nuclear PTEN expression was weakly associated with time to relapse, and this did not reach significance. Nuclear PTEN expression was, however, independently strongly associated with overall survival, and the curves on the Kaplan–Meier plot begun to separate almost immediately after diagnosis. These results in combination with the lack of correlation with Akt activation suggest that the role of PTEN in the nucleus is independent of cytoplasmic PTEN. It is now recognised that PTEN has a function in the nucleus, and reports of PTEN nuclear localisation have begun to multiply over the past few years in tumour and non-tumour cells (Gimm *et al*, 2000). Chung *et al* (2005) have demonstrated that PTEN has dual nuclear localisation signal-like sequences that mediate nuclear import, and have shown that nuclear PTEN is required for PTEN-mediated cell-cycle arrest and growth inhibition through the downregulation of cyclin D1. Reports from the literature also suggest that PTEN localises to the nucleus during the G_0_-G_1_ phase of cell cycle and mediates growth suppression through the inhibition of MAP kinase phosphorylation independent of Akt activation (Chung and Eng, 2005; Chung *et al*, 2006). Results from our study support the hypothesis that PTEN has a distinct function in the nucleus independent of its cytoplasmic role.

However, it is a possibility that nuclear PTEN is simply a surrogate marker of PTEN activation as *in vitro* studies demonstrate that following phosphorylation, PTEN is released from the membrane-bound scaffolding proteins and enters the nucleus. In support of this, we report a correlation between membrane and nuclear PTEN expression (*P*<0.001, Rs 0.66), and PTEN membrane expression is also linked to survival. However, in contrast to cytoplasmic PTEN expression, no correlations were observed between nuclear PTEN expression and Akt activation; therefore, the evidence to support nuclear PTEN as a surrogate marker of PTEN cytoplasmic activation is not convincing in this study.

In summary, the rate of loss of *PTEN* as measured by FISH increased with disease progression, and a trend was noted between *PTEN* loss and poorer disease-specific survival, suggesting that this arm of the study should be expanded to larger cohort. In addition, both cytoplasmic and nuclear PTEN are independently associated with good outcome measures in hormone-sensitive prostate cancer, but appear to have independent roles.

## Figures and Tables

**Table 1:** Histoscore variation and comparison of staining intensity for hormone-sensitive and hormone-refractory tumours

	**HSPC (IQR)**	**HRPC (IQR)**	***P*-value**	**ICCC**	**Fallers (%)**	**Risers (%)**
**PTEN** membrane	0–67.5	0–40	0.086	0.84	33	15
**PTEN** cytoplasm	80–150	80–107	0.104	0.90	33	23
PTEN **nucleus**	0–50	0–80	0.588	0.82	28	25

The interquartile range (IQR) for hormone-sensitive tumours (HSPC) and hormone-refractory tumours (HRPC) are shown. The *P*-value was compared using a Wilcoxon signed-rank test. The interclass correlation coefficient (ICCC), which measures the consistency among observers for each protein, is consistently higher than 0.7, which is classed as excellent. The percentage of tumours that was defined as having a rise or fall in protein expression (calculated using the number of histoscore units that is defined as a change in expression) is also shown.
